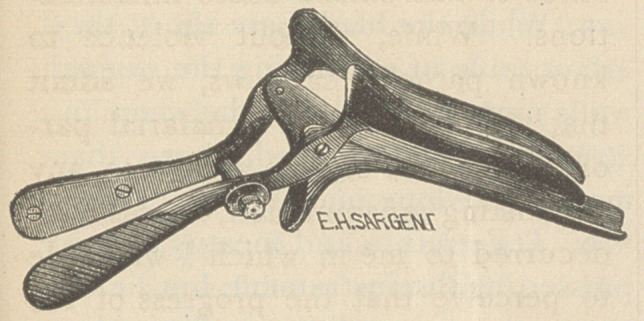# An Improved Speculum

**Published:** 1874-09-01

**Authors:** Daniel T. Nelson

**Affiliations:** Prof. Physiology and Histology Chicago Medical College; 1108 Indiana avenue


### AN IMPROVED SPECULUM.

By Daniel T. Nelson, M.D., Prof. Physiology and Histology Chicago Medical College.

So many forms of uterine speculum are now to be found in the stores, one may well hesitate to add to the number—already legion. But this variety demonstrates both the progress of gynecology and the probable fact that a perfect speculum has not yet been made.

The one I now offer the profession is very well represented by the accompanying wood-cut.

As will be seen, it is more like Nott's latest than any other instrument. It differs from Nott's in having the lower blade longer and of better shape to receive the neck of the uterus, and in having handles for elevating and holding the upper blades.

The measurements of the instrument are as follows: Lower blade, $4\frac{1}{2}$ inches; extending beyond upper blades 5-8 of an inch; length of instrument, including handles, $7\frac{1}{2}$ inches. The upper blades are made shorter than the lower to correspond with the anatomy of the parts, as the posterior vaginal wall is longer than the anterior.

Some object to Nott's, and doubtless will to this instrument, that it is too short. But no physician has any difficulty in reaching the os uteri, except in rare cases, with the index finger, the available length of which rarely exceeds three and one-half inches, and the lower blade of my instrument is four and one-half inches in length and the upper blades nearly four inches. If the os is not exposed when the instrument is expanded, the difficulty is not in the length of the instrument but in its position, or because it is not sufficiently expanded to raise the anterior wall of the vagina.

To introduce the instrument: The patient reclines on the back upon the gynæcological chair, with the hip near the edge of the chair. Having ascertained the position of the os uteri, grasp the speculum with the right hand with the fore-finger resting upon and projecting beyond the lower blade, and hold the handles vertical. Then carefully introduce the fore-finger into the external organs and follow it with the instrument. When the instrument has passed beyond the external organs, it should be rotated so the handles shall lay horizontally; then, pushing the lower blade along the posterior wall of the vagina, it will pass under the posterior labium of the os. Then, compressing and bearing downwards and backwards upon the handles, the anterior vaginal wall will be raised and the os exposed, when the handles can be fastened by the thumb-screw. The instrument is self-retaining when sufficiently expanded.

If the os is not at first exposed, the instrument, partially expanded, may be withdrawn a little so as to allow the lower blade to pass under the os; or the os may be raised by the fore-finger inserted through the expanded instrument, by raising the anterior wall of the vagina, there being ample room for the fore-finger to pass between the expanded upper blades. Or the os may be raised into the field of the instrument by a Simpson's sound, or like instrument, used as a lever. When the os is exposed, the uterus may be held in the field by a tenaculum, which can be fastened to a hook on the right upper blade.

My tenaculum is the same as Nott's, except that it has a handle like an applicator. When the tenaculum is fastened into the anterior labium of the os from below upward, it rarely is felt at all by the patient, and the little hæmorrhage which may occur will be of no disadvantage. The advantages claimed for this speculum are:

1. Its length is such as to expose the uterus *in situ* by bringing it *nearer* the external organs, rather than pressing it deeper into the pelvis as do the longer instruments.

2. Thus giving a *better light*, which is often of great importance, especially when the physician is obliged to visit the patient at her home.

3. The instrument is so short, and the upper blades expand in such a manner as to readily allow of the rectifying of any malpositions of the uterus through the expanded instrument, which is impossible in all the long instruments.

4. A large portion of the *vaginal walls* are exposed for examination and treatment, if needed, and by rotating the instrument the whole may be exposed.

5. While the blades are short, they are capable of expanding the vaginal walls more than any of the short instruments, and, indeed, more than most of the long ones.

6. The urethra and meatus are not pressed by the instrument, but lie between the upper blades, where they may be readily examined and treated if necessary.

I am under obligations to Mr. E. H. Sargent for the mechanical beauty and perfection of the instrument, and for the interest he has taken in its success.

The speculum may be seen at Sargent's, 785 Wabash ave., cor. Sixteenth St., Chicago, and at Codman & Shurtleff's, Boston, Mass.

1108 Indiana avenue.

UNIVERSITY OF VIENNA.—It is announced that Prof. Rokitansky is about to retire from the chair of Pathology in this University. Prof. Von Recklinghausen, of Strasburgh, has been invited to become his successor.—*Med. News.*

## Figures and Tables

**Figure f1:**